# Medium-Chain Acyl-CoA Deficiency: Outlines from Newborn Screening, *In Silico* Predictions, and Molecular Studies

**DOI:** 10.1155/2013/625824

**Published:** 2013-10-31

**Authors:** Serena Catarzi, Anna Caciotti, Janita Thusberg, Rodolfo Tonin, Sabrina Malvagia, Giancarlo la Marca, Elisabetta Pasquini, Catia Cavicchi, Lorenzo Ferri, Maria A. Donati, Federico Baronio, Renzo Guerrini, Sean D. Mooney, Amelia Morrone

**Affiliations:** ^1^Department of Neurosciences, Psychology, Pharmacology and Child Health, University of Florence, Viale Pieraccini 24, 50139 Florence, Italy; ^2^Molecular and Cell Biology Laboratory, Paediatric Neurology Unit and Laboratories, Neuroscience Department, A. Meyer Children's Hospital, Viale Pieraccini 24, 50139 Florence, Italy; ^3^Buck Institute for Research on Aging, 8001 Redwood Blvd., Novato, CA 94945, USA; ^4^Newborn Screening Biochemistry and Pharmacology Laboratory, Clinic of Paediatric Neurology, A. Meyer Children's Hospital, Viale Pieraccini 24, 50139 Florence, Italy; ^5^Metabolic Disorders Unit, Neuroscience Department, A. Meyer Children's Hospital, Viale Pieraccini 24, 50139 Florence, Italy; ^6^Department of Pediatrics, University of Bologna, Via Massarenti 13, 40138 Bologna, Italy; ^7^Pediatric Neurology Unit and Laboratories, Neuroscience Department, A. Meyer Children's Hospital, Viale Pieraccini 24, 50139 Florence, Italy

## Abstract

Medium-chain acyl-CoA dehydrogenase deficiency (MCADD) is a disorder of fatty acid oxidation characterized by hypoglycemic crisis under fasting or during stress conditions, leading to lethargy, seizures, brain damage, or even death. Biochemical acylcarnitines data obtained through newborn screening by liquid chromatography-tandem mass spectrometry (LC-MS/MS) were confirmed by molecular analysis of the medium-chain acyl-CoA dehydrogenase (*ACADM*) gene. Out of 324.000 newborns screened, we identified 14 MCADD patients, in whom, by molecular analysis, we found a new nonsense c.823G>T (p.Gly275∗) and two new missense mutations: c.253G>C (p.Gly85Arg) and c.356T>A (p.Val119Asp). Bioinformatics predictions based on both phylogenetic conservation and functional/structural software were used to characterize the new identified variants. Our findings confirm the rising incidence of MCADD whose existence is increasingly recognized due to the efficacy of an expanded newborn screening panel by LC-MS/MS making possible early specific therapies that can prevent possible crises in at-risk infants. We noticed that the “common” p.Lys329Glu mutation only accounted for 32% of the defective alleles, while, in clinically diagnosed patients, this mutation accounted for 90% of defective alleles. Unclassified variants (UVs or VUSs) are especially critical when considering screening programs. The functional and pathogenic characterization of genetic variants presented here is required to predict their medical consequences in newborns.

## 1. Introduction

Medium-chain acyl-CoA dehydrogenase deficiency (MCADD) is the most common metabolic defect of fatty acid oxidation. MCAD (MCAD E.C. 1.3.99.3) is a mitochondrial flavoprotein which catalyzes the first reaction in *β*-oxidation of fatty acids with medium-chain length [1]. The enzymatic defect results in a decrease of ketone production as well as an increased concentration of medium-chain fatty acids. The breakdown of such lipids is essential for energy production during periods of prolonged fasting or physiological stress. Illnesses associated with gastrointestinal symptoms such as lost appetite with vomiting and diarrhea can precipitate an acute metabolic crisis in affected individuals, resulting in the accumulation of potentially toxic acylcarnitine, hypoketotic hypoglycemia, Reye syndrome-like episodes, seizures, brain damage, and death, including sudden unexpected death in infancy [2–4]. If undetected, approximatively 20%–25% of patients die during their first metabolic crisis or suffer developmental delay and permanent neurologic impairment [5].

Preventive measures, including avoidance of fasting and rapid treatment of catabolic stress, have been shown to reduce morbidity and mortality [5–7].

MCAD deficiency is inherited as an autosomal recessive trait and is caused by mutations in the medium-chain acyl-CoA dehydrogenase (*ACADM*) gene, which is located on chromosome 1p31 and consists of 12 exons spanning 44 Kb [8]. The active enzyme has a homotetrameric form [9–11]. Each subunit of MCAD enzyme is composed of three structural domains: the N-terminal *α*-helix domain (residues 1–129), the *β*-sheet domain (residues 130–239), and the C-terminal *α*-helix domain (residues 240–396). The N- and C-terminal domains consist mainly of tightly packed *α*-helices that form the tetramer core. The middle *β*-domains are exposed at the surface of the molecule and comprise two orthogonal *β*-sheets. The catalytic sites consist of the binding sites for the substrate and the natural cofactor flavin adenine dinucleotide (FAD) and are mainly formed by the interface between the *β*-domain and the C-terminal *α*-domain [12]. 

Newborn screening (NBS) for MCAD deficiency has recently been implemented worldwide using liquid chromatography-tandem mass spectrometry (LC-MS/MS) to analyze blood spots from newborns for acylcarnitines thus making the identification of asymptomatic patients and the identification of a much wider spectrum of genetic lesions in the *ACADM* gene possible [5, 13–16].

To date, more than 90 *ACADM* gene mutations have been described, with most being missense (HGMD Professional Database: http://www.biobase-international.com/product/hgmd). The most common mutation is the c.985A>G (p.Lys329Glu) change, which in MCADD patients of European descent, is observed at the homozygous state in 80% and at the heterozygous status in about 18%. The remaining 2% of MCADD patients carry other rare mutant alleles [17, 18].

Here, we report biochemical and genetic studies on MCADD neonates identified through NBS by LC-MS/MS performed on the whole newborns of central Italy (Tuscany and Umbria regions).

## 2. Methods

### 2.1. Patients

Patients included in this study came to our attention to confirm a biochemical suspicion of MCAD deficiency as a consequence of abnormal NBS results. Patients (Pts) reported here were twelve of Italian origin and two (Pt12 and Pt13) of Albanian origin. All patients were unrelated, and Pt12 and -13 were found to be consanguineous.

### 2.2. Biochemical Analysis

Blood acylcarnitines from newborns dried blood spots (DBSs) were quantified by LC-MS/MS [25].

### 2.3. Genomic DNA Analyses

Molecular studies were performed after receiving informed consent for genetic testing. Genomic DNA was obtained from patients' lymphocytes using QIAsymphony instrument as recommended by the manufacturer (Qiagen, Hilden, Germany). The minimum amount of requested whole blood for each DNA extraction was 1.3 mL. The entire *ACADM *coding region and exon/intron boundaries were amplified using previously described primers and conditions [26], and purified PCR products were directly sequenced on ABI PRISM 3130 XL Genetic Analyzer using Big Dye Terminator chemicals (Applied Biosystems, Foster City, CA, USA).

### 2.4. Screening of New Mutations and Bioinformatics Analysis

The *ACADM* gene of 80 healthy control DNA samples was analyzed by sequencing analysis of the fragments containing the new missense mutations identified. Moreover, these new mutations were examined in the recently available 1000 Genomes Project database (http://browser.1000genomes.org/index.html). In addition, multiple sequence alignment (MSA) of *ACADM-*related proteins was performed using Muscle [27], and the MSA was visualized and sequence conservation was analyzed by ConSurf [28]. The possible impact of novel amino acid substitutions on MCAD structure and function was evaluated by MutPred [29]. The effects of mutations on protein stability were calculated by I-Mutant 2.0 [30].

### 2.5. Structural Analyses

To predict the structural effect of the novel missense mutations on the resulting MCAD enzymes, we visualized the mutations on the three-dimensional structure of MCAD based upon the crystal structure of the human isoform (PDB: 1EFE) [31]. Mutation positions were visually inspected by UCSF Chimera (ref. Pubmed ID 15264254) for changes in structural properties, functional regions (such as ligand binding), and electrostatics.

## 3. Results

### 3.1. Biochemical Analysis

The results of metabolite analyses are given in Table 1. Blood acylcarnitine profile in affected patients showed elevations of medium-chain acylcarnitines (from C6 to C10) with predominance of octanoylcarnitine (C8). In our data, C8/C6 and C8/C10 ratios were also significantly elevated.

### 3.2. Molecular Characterization and *In Silico* Analysis

The patients' *ACADM* gene coding regions and the correspondent exon/intron boundaries were amplified and directly sequenced on both strands. Molecular data on all fourteen MCADD patients identified in our unit since 2002 are summarized in Table 1. All identified mutations were confirmed in the parents' genomic DNA, and all at-risk family members were also screened.

Three new *ACADM* nucleotide variants leading to two new amino acid substitutions c.253G>C (p.Gly85Arg) and c.356T>A (p.Val119Asp) and a new nonsense mutation c.823G>T (p.Gly275*) were identified.

The absence of the genetic lesions leading to the new missense mutations in 160 control alleles and their absence in the 1000 Genomes Project database suggest that their incidence is <1% in the normal population consistent with a possible pathogenetic role of the identified genetic lesions. Both missense mutations are located in conserved positions in the sequence alignment of 11 human MCAD-related proteins. MutPred predicted all of the two mutations to be damaging, with a score of 0.835 for p.Val119Asp and 0.933 for p.Gly85Arg. MutPred gives the mutations a probability score that ranges from 0 to 1 by MS/MS (mutations with scores >0.5 are considered likely pathogenic), so p.Val119Asp and p.Gly85Arg have especially high probability of pathogenicity.

### 3.3. Three-Dimensional Analyses

**Figure 1:**
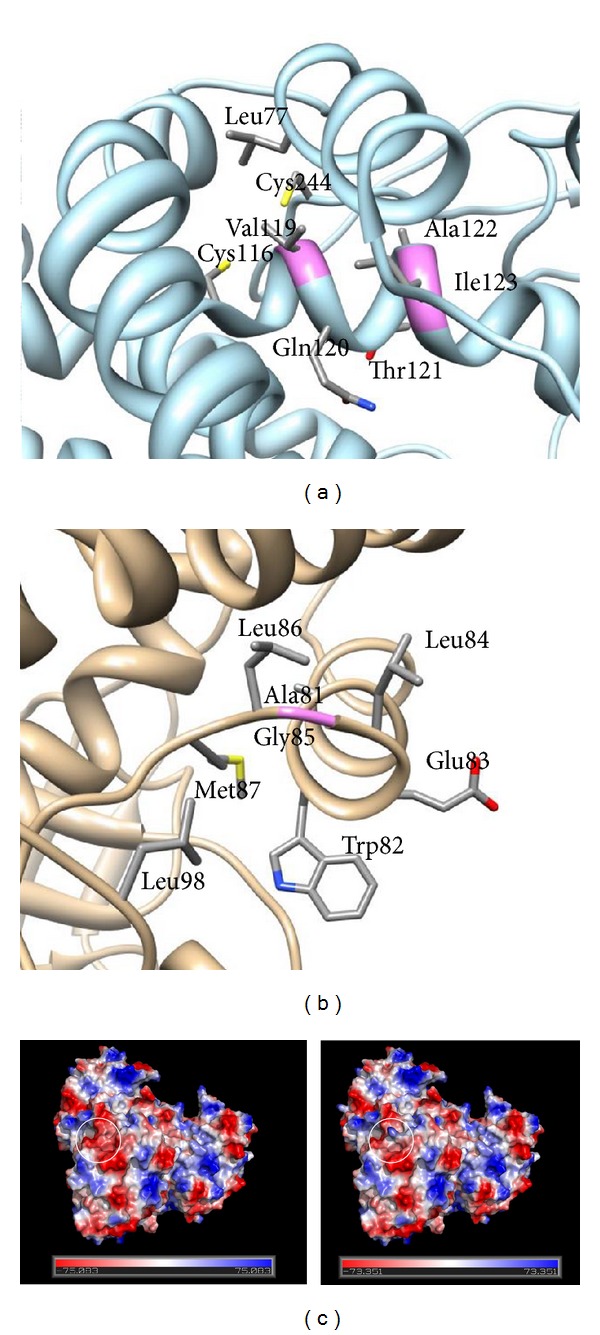
(a), (b) MCAD three-dimensional structure highlighting positions of the mutations. PDB structure 1EGE (ref. Pubmed ID 8823176) was downloaded and visualized in UCSF Chimera (ref. Pubmed ID 15264254). Sidechains of all amino acids with heavy atoms within 4 angstroms were displayed on a ribbon backbone. In (a), valine 119 is shown in its environment, whereas (b) shows glycine 85 in its environment. The positions of the mutations are shown in violet. (c) Electrostatic surface potential of the wild-type form (on the left) and of the p.Gly85Arg mutant form (on the right). The electrostatic surface potential is indicated in red (negative charge), white (uncharged), and blue (positive charge). The figure is generated using the PyMOL Molecular Graphics System, Version 1.5.0.4 Schrödinger, LLC.

To further elucidate the effects of the new amino acid changes, we interrogated the mutant MCAD structures (Figures 1(a) and 1(b)). The mutation p.Gly85Arg is not positioned in ligand-binding or catalytic residues. 

p.Val119 is located in an alpha helix further away from the catalytic site. The p.Val119Asp mutation likely destabilizes the protein structure, because the wild-type residue is hydrophobic and buried in the protein structure, while the mutant residue (Asp) brings charge to the hydrophobic environment where residue 119 is located. Also, p.Val119 only makes hydrophobic contacts (calculated with CSU [32]). The program I-Mutant 2.0 [30] also predicts this mutation to be destabilizing. There is no notable change in the surface electrostatics even though the mutation causes a local charge change.

p.Gly85Arg is located in the loop right after helix 3, which is far apart from the catalytic site, on the opposite surface of the protein. The mutation causes a charge change, and also the mutation causes an amino acid change from no sidechain to a long and bulky sidechain. The large sidechain can be accommodated in the structure; however, because the residue is positioned on the protein surface, p.Gly85Arg causes a change in the electrostatic surface potential of the more positive protein, while the more favorable conformational flexibility of the glycine backbone is perhaps further stabilizing (Figure 1(c)).

None of the missense mutations is located at the electron transfer flavoprotein binding surface, but residues after 275 are. Therefore, p.Gly275P* might cause loss of electron transfer from MCAD.

## 4. Discussion

The risk of sudden death or severe and persistent neurological damage in undiagnosed MCADD patients and the possibility of treating this metabolic disorder with simple dietary measures were the reasons driving the expansion of newborn screening using LC-MS/MS. 

This methodology has been successfully implemented worldwide thus revealing a higher incidence of MCADD than clinically suspected, making it the most frequently diagnosed disease using DBSs, alongside phenylketonuria [5, 14–16].

Indeed, before the advent of NBS using LC-MS/MS, the clinically ascertained incidence of MCADD was roughly 1 : 30,000 to 1 : 135,000 [13, 33], while population-based NBS is revealing a much higher incidence of MCADD in newborns, ranging from 1 in 10,000 to 1 in 20,000 in different populations [19, 20, 33–37]. In particular, based on newborn screening programs worldwide, the highest incidence of MCADD seems to be in Northern Germany (about 1 : 5000) and the lowest in Far East populations (Japan and Taiwan) [38–40].

In our laboratory, the neonatal screening program based on expanded LC-MS/MS-NBS has been performed since 2002 in central Italy. After screening 324,000 newborns, we uncovered 14 MCAD deficiency cases. These findings reveal an increased rate of diagnosis of 1 : 23,000, in agreement with published data.

MCADD undiagnosed individuals are asymptomatic until an episode of increased energy demand and fasting occurs, resulting in metabolic crisis or even sudden death. Prior to the advent of expanded newborn screening, sudden and unexplained death was often the first and only occurrence of MCADD [3, 41, 42].

The great potential of NBS by LC-MS/MS is to identify asymptomatic patients thus allowing newborns preventive care that may prevent crises and neurological damage. Molecular analysis of *ACADM* gene in newborns with altered acylcarnitines profile can provide appropriate genetic counseling as well as prenatal diagnosis to affected families.

The importance of early diagnosis is confirmed by the observation that during followup none of our patients suffered episodes of acute metabolic decompensation, likely due to the preventive role of avoiding fasting and by intravenous glucose therapy in the course of infectious or gastrointestinal episodes.

Among our MCADD cohort patients, we found three new *ACADM* mutations: two missense c.253G>C (p.Gly85Arg) and c.356T>A (p.Val119Asp), and one nonsense c.823G>T (p.Gly275*), which contribute to delineate the molecular genetic heterogeneity of MCADD. As described in Table 1, we identified 14 MCADD patients overall in our unit. Out of the 28 mutated alleles they carried, only 9 are represented by the common p.Lys329Glu mutation (32% of defective alleles), which we detected at the homozygous level in three newborns and at heterozygous level in the remaining three. p.Lys329Glu accounts for 90% of defective alleles in patients diagnosed after metabolic decompensation [43]. Examples of reference data on acylcarnitine thresholds for NBS are available from the National Institute for Public Health and Environment of the Netherlands [44]. The *ACADM* gene sequencing analysis can help to discriminate healthy heterozygous carriers from affected individuals [45]. However, the validation of MCADD newborn screening programs, that is, cutoff policies, and the natural course of milder affected neonates have been strongly discussed in the recent years [44–50]. Insights on risk assessment and counseling of patients have been proposed by mapping mutations onto structural models [51] and by the evaluations of stability and enzyme kinetics [46, 48, 51]. However, unless subjects with MCAD enzyme activities >10% were proposed to be considered as normal individuals, emergency regimen and parental instructions remain necessary also for these subjects [50]. 

All our 14 patients are still asymptomatic; thus, bioinformatics analysis may be particularly useful for the characterization of the new mutations. This type of analysis showed that p.Gly85 is conserved among species, suggesting that replacement of this residue has a significant fallout on enzyme functionality.

p.Val119 is not conserved among species (data not shown), but its substitution to Asp is a major amino acid change which is also predicted by three-dimensional analysis as destabilizing. Since in Pt2 the other allele is a null allele, p.Val119 amino acid change certainly affects MCAD protein function. The severity of the p.Val119Asp can also be estimated comparing the blood content of acylcarnitines in Pt2 with that of Pt4 who carries two null alleles: C8-acylcarnitine level in Pt2 was indeed even higher than that of Pt4.

In this regard, it has been demonstrated that patients with MCADD are at risk of a symptomatic episode regardless of their genotype or of the initial C8 level on NBS. Neither genotype nor metabolite levels protect from a potentially poor outcome. However, the significantly higher NBS C8 level in patients reporting symptomatic episodes suggests that neonates having high initial C8 levels may exhibit a reduced ability to sustain later metabolic stress. These infants more likely carry severe mutations as deletions, nonsense, or splice defects and the common p.Lys329Glu mutation [46]. However, it must be considered that during the first hours of life the level of blood acylcarnitines may also vary due to the extent of neonatal weight loss, to the possible administration of glucose-containing solutions, and to the time between the birth of the baby and the occurrence of breast milk. These events may retard the occurrence of fasting and hypoglycemia and explain the wide range of blood acylcarnitines (C8 and C8/C10 levels) in affected patients even if such levels are compared in different patients (Pt12 and Pt14) carrying the same (c.985A>G, p.Lys329Glu) *ACADM* gene mutation.

The three new alleles we identified are correlated with high (p.Gly85Arg) or very high levels (p.Val119Asp and p.Gly275*) of C8-carnitines; it is likely that they can be defined as severe mutations, as our *in silico* analysis confirmed. 

## 5. Conclusions

 Our data confirm the high incidence of MCADD due to the sensibility and reliability of acylcarnitines analysis by LC-MS/MS analysis, making possible early specific therapies that can prevent possible crises in at-risk infants.

NBS by LC-MS/MS is revealing a wider spectrum of *ACADM* mutations than what had previously emerged from molecular investigations of clinically ascertained patients. 

Molecular studies supported by *in silico* analysis can be important to confirm the MCADD diagnosis. Along with deepening the pathophysiology of MCAD deficiency, the evaluation of the natural course of milder variants will serve to provide or integrate a general model of MS/MS-based newborn screening program.

## Figures and Tables

**Table 1:** Newborn screening acylcarnitines results and molecular analysis of MCADD patients identified by LC-MS/MS NBS since 2002.

Patient	C8	C6	C10	C10:1	C8/C6	C8/C10	Nucleotide changes	Mutations	Mutation references
1	4.02	0.67	0.39	—	6	10.3	**c.253G>C/c.253G>C**	**p.Gly85Arg/p.Gly85Arg**	This work/this work
2	15.36	1.85	1.72	10.33	8.3	8.93	**c.356T>A**/c.244dupT	**p.Val119Asp**/p.Trp82Leufs*23	This work/[19]
3	3.87	0.79	1.13	0.42	4.89	3.34	c.728G>A/c.199T>C	p.Arg243Gln/p.Tyr67His	[20]/[21]
4	9.5	1.15	0.83	0.64	8.26	11.44	**c.823G>T/c.823G>T**	p.Gly275*/p.Gly275*	This work/this work
5	41.18	2.01	3.59	0.95	20.48	11.47	**c.356T>A**/c.946-2A>C	**p.Val119Asp**/IVS10-2A>C	This work/[7]
6	13.42	1.44	1.14	0.48	9.32	11.77	c.985A>G/c.431_434delAGTA	p.Lys329Glu/p.Lys144Ilefs*5	[22]/[23]
7	1.3	0.32	0.87	0.55	4.06	1.49	c.985A>G/c.127G>A	p.Lys329Glu/p.Glu43Lys	[22]/[23]
8	6.96	1.24	1.26	0.54	5.61	5.52	c.985A>G/c.799G>A	p.Lys329Glu/p.Gly267Arg	[22]/[24]
9	8.79	0.76	0.69	0.5	11.56	12.73	c.985A>G/c.985A>G	p.Lys329Glu/p.Lys329Glu	[22]/[22]
10	9.77	0.9	0.82	0.72	10.85	11.91	c.985A>G/c.985A>G	p.Lys329Glu/p.Lys329Glu	[22]/[22]
11	26.7	3.35	3.03	0.9	7.97	8.81	c.985A>G/c.985A>G	p.Lys329Glu/p.Lys329Glu	[22]/[22]
12	15.4	2.2	0.98	1.33	7	15.71	c.244dupT/c.244dupT	p.Trp82Leufs*23/p.Trp82Leufs*23	[19]/[19]
13	25.1	2.53	2.41	1.48	9.92	10.41	c.244dupT/c.244dupT	p.Trp82Leufs*23/p.Trp82Leufs*23	[19]/[19]
14	0.75	0.18	0.04	0.35	4.16	18.75	c.244dupT/c.244dupT	p.Trp82Leufs*23/p.Trp82Leufs*23	[19]/[19]

Acylcarnitines normal values: C8 < 0.31 *μ*mol/L, C6 < 0.25 *μ*mol/L, C10 < 0.36 *μ*mol/L, C10:1 < 0.50 *μ*mol/L, C8/C6 0.85–3 *μ*Mmol/L, and C8/C10 0.33–1.6 *μ*Mmol/L.

*ACADM* gene reference sequence NM_000016.4; in bold characters are indicated the new mutations found.

## Abbreviations

C8: Octanoylcarnitine
DBS: Dried blood spot
LC-MS/MS: Liquid chromatography-tandem mass spectrometry
MCADD: Medium-chain acyl-CoA dehydrogenase deficiency
NBS: Newborn screening
Pt: Patient.
